# Senolytic Cocktail Dasatinib+Quercetin (D+Q) Does Not Enhance the Efficacy of Senescence-Inducing Chemotherapy in Liver Cancer

**DOI:** 10.3389/fonc.2018.00459

**Published:** 2018-10-30

**Authors:** Kristina Kovacovicova, Marianna Skolnaja, Mihkel Heinmaa, Martin Mistrik, Pille Pata, Illar Pata, Jiri Bartek, Manlio Vinciguerra

**Affiliations:** ^1^International Clinical Research Center (FNUSA-ICRC), Brno, Czechia; ^2^Department of Chemistry and Biotechnology, Tallinn University of Technology, Tallinn, Estonia; ^3^IVEX Lab, Tallinn, Estonia; ^4^Faculty of Medicine and Dentistry, Institute of Molecular and Translational Medicine, Palacky University, Olomouc, Czechia; ^5^Genome Integrity Unit, Danish Cancer Society Research Center, Copenhagen, Denmark; ^6^Science for Life Laboratory, Division of Translational Medicine and Chemical Biology, Department of Medical Biochemistry and Biophysics, Karolinska Institute, Solna, Sweden; ^7^Division of Medicine, Institute for Liver and Digestive Health, University College London, London, United Kingdom

**Keywords:** NAFLD (non-alcoholic fatty liver disease), HCC (hepatocellular carcinoma), senolytic agents, quercertin, dasatinib

## Abstract

Hepatocellular carcinoma (HCC) is a leading cause of cancer-related death, which develops in the context of fibrosis and cirrhosis caused by chronic inflammation, in turn due to non-alcoholic fatty liver disease (NAFLD), alcohol consumption and/or hepatitis viral infection. An increased number of senescent cells are associated with age-related tissue degeneration during NAFLD-induced HCC, or during chemotherapeutic treatment. Senolytic agents target selectively senescent cells. A combination of the senolytic drugs dasatinib and quercetin (D+Q) reduced hepatic lipid accumulation and alleviated age-associated physical dysfunction in mice. However, whether D+Q can impact the treatment of HCC, at the end-stage of the NAFLD inflammatory spectrum, is unknown. Here, using two well-established HCC cell lines (HepG2, Huh-7), we demonstrate that the maximal cytostatic doses for D and/or Q (1 + 1 μM) lacked efficacy in removing doxorubicin-induced β-gal-positive senescent cells. Moreover, D+Q did not affect doxorubicin-dependent induction of flattened morphology, activation of p16, expression of SASP-associated genes or formation of γH2AX foci. We then investigated the antitumor efficacy of doxorubicin, D+Q, or the combination, in xenograft studies conducted with HCC cells inoculated in athymic nude mice. Doxorubicin reduced tumor growth by 30% compared to control mice, while D+Q was ineffective in synergizing with doxorubicin and in clearing doxorubicin-induced HCC senescent cells. Unexpectedly, D+Q alone appeared to have acute pro-tumorigenic effects in control mice. While our data need to be confirmed in animal models that fully recapitulate NAFLD, we demonstrate that these compounds are ineffective, alone or in synergy with senescence-inducing chemotherapy, against experimental HCC.

## Introduction

Hepatocellular carcinoma (HCC) is the second leading cause of cancer-related death in the world (1). As a result of advanced disease or poor liver function, few patients with HCC are eligible for surgery: the leading chemotherapeutics agents (sorafenib, doxorubicin, and others) do not offer an increase in the average survival time of 6 months (1–3). Most HCCs develop in the context of liver steatosis, fibrosis and cirrhosis caused by chronic inflammation. These disturbances can be part of a clinical spectrum called non-alcoholic fatty liver disease (NAFLD), or of prolonged alcohol abuse and/or hepatitis viral infection. Regardless of the etiology, disease progression to HCC takes several decades and is thus accrued by aging: in a recent US study enrolling 59,907 the median age at the time of diagnosis of HCC was 62 years (4). Cellular senescence refers to a state of stable cell-cycle arrest combined with the senescent associated secretory phenotype (SASP) (5). Great hopes rely on the so called senolytics, agents that selectively induce removal of senescent cells, and alleviate multiple age-related phenotypes (6). An increased number of senescent cells is associated to age-related tissue degeneration during NAFLD-induced HCC (7, 8). Ogrodnik et al. showed that treatment with a combination of the senolytic drugs dasatinib and quercetin (D+Q) leads to an overall reduction of NAFLD in aged dietary-induced NAFLD mice models (9). Xu et al. recently showed in a ground breaking report that oral administration of D+Q to naturally aged mice alleviated physical dysfunction and increased their survival by 36% (10). D+Q previously showed efficacy also in pre-clinical models of fibrotic pulmonary disease (11). If D+Q have an effect also on the treatment of the more serious HCC condition, at the end of the liver disease inflammatory spectrum, is unknown.

## Results and discussion

**Figure 1:**
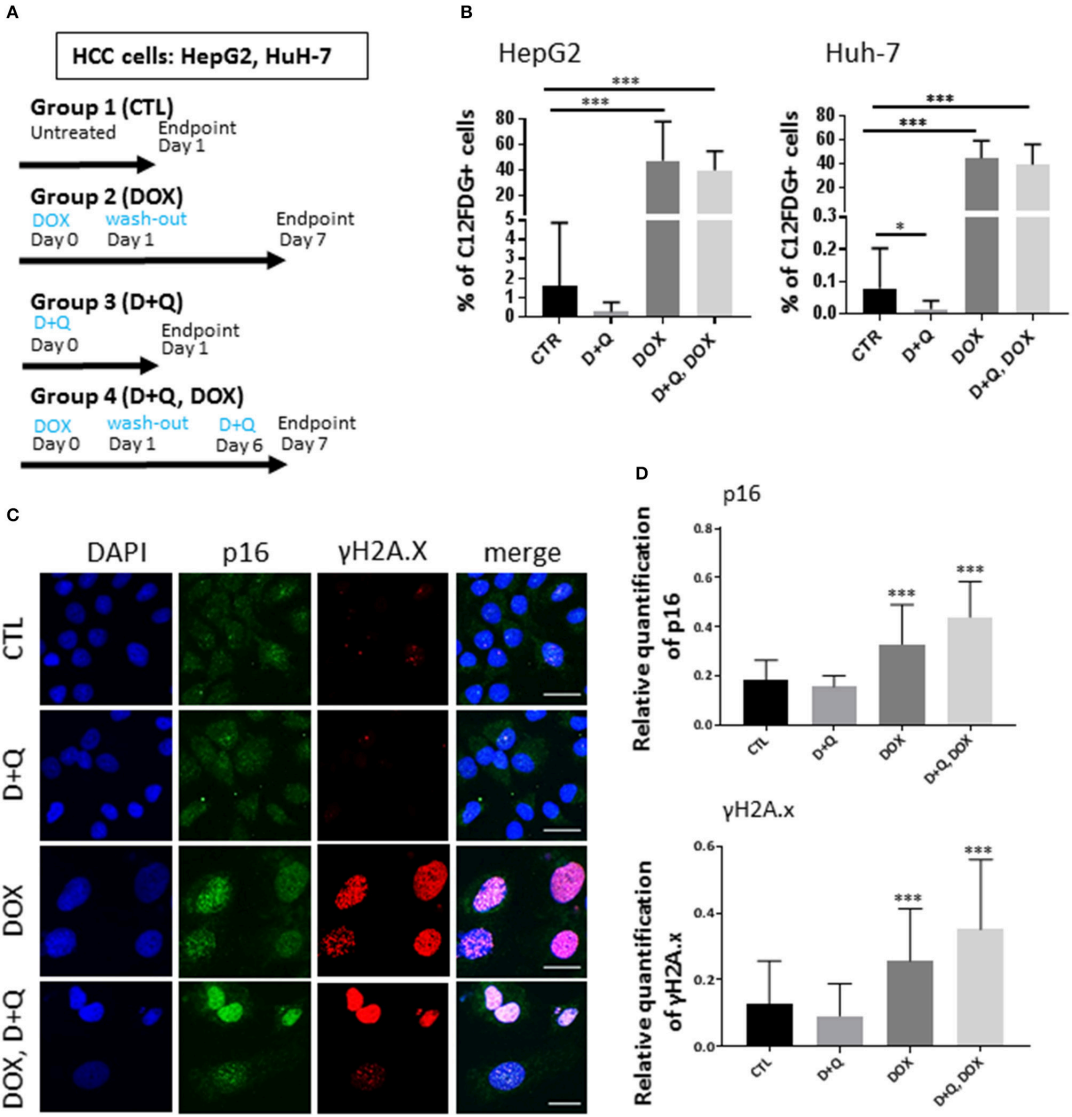
Effect of D+Q on chemotherapy-induced senescence of HepG2 and Huh-7 HCC cells. **(A)** Four experimental groups of either HepG2 or Huh-7 HCC cells were created: 1. CTL; 2. DOX; 3. D+Q; 4. D+Q, DOX. See main text for details. **(B)** CTL; D+Q; DOX and D+Q, DOX-treated HepG2 or Huh-7 cells were incubated with C_12_FDG. *N* = 3. **(C)** Left panels: representative micrographs displaying DAPI, p16, and γH2A.X in control, DOX-treated, D+Q-treated or D+Q, DOX-treated HuH-7 cells; Right panels: quantification of p16 staining intensity or of γ-H2A.X positive cells. **(B,D)**, ^*^p < 0.05; ^***^p < 0.001 compared to CTL.

**Figure 2:**
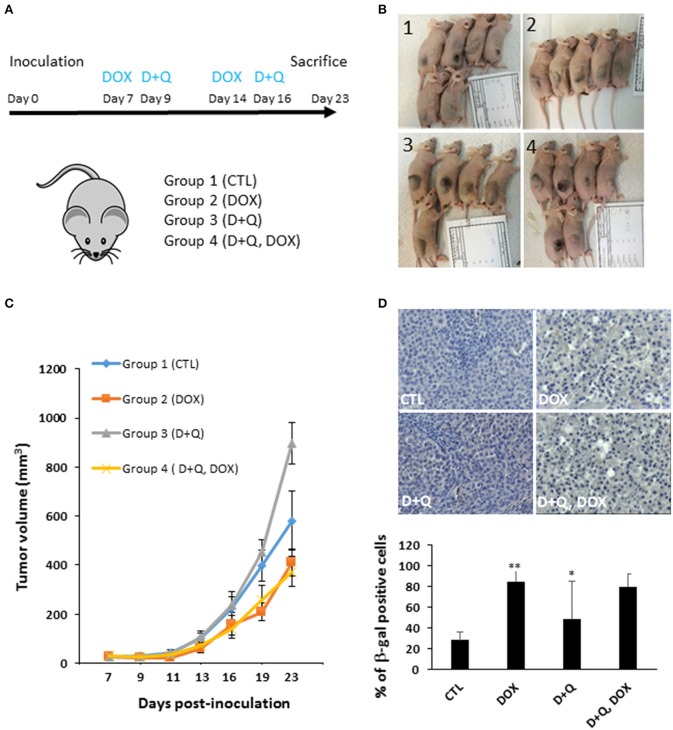
Effect of D+Q on chemotherapy-induced decrease in tumor growth and senescence *in vivo*. **(A)** Four experimental groups of balb/c nude mice (*n* = 11 per group) implanted with Huh-7 cells were created: 1. CTL; 2. DOX; 3. D+Q; 4. D+Q, DOX. See main text for details. **(B)** Representative images of mice at euthanasia. **(C)** Time-dependent tumor volume assessment by caliper. Statistical differences between the experimental groups at day 23 are shown. **(D)** representative pictures of SA-β-gal immunostaining of tumor sections from mice as in **(A)** (*n* = 3). The percentage of SA-β-gal-positive cells was calculated in 10 blindly chosen at a magnification of ×200. D, ^*^p < 0.05; ^**^p < 0.01; compared to CTL.

To address this issue, first we tested the separate dose-dependent effects of D or Q on the viability of two well-established HCC cell lines (HepG2, Huh-7) (7, 12–16); we administered D or Q to increased concentrations of D or Q (1 nM−1 mM) to HepG2 and Huh-7 cells and identified 1 μM as the maximal cytostatic dose for either drug, without displaying cytotoxic effects (Figure S1A). Subsequently, we tested the impact of combined D+Q on the cellular senescence induced by the chemotherapeutic agent doxorubicin, according to scheme in Figure 1A. Four experimental groups were considered: (1) control cells; (2) cells treated with doxorubicin (DOX) at 100 nM, a concentration slightly cytotoxic in HCC cells (15, 17, 18). DOX was washed out after 24 h and cells were incubated in normal medium for seven additional days, before analyses; (3) D+Q, cells were treated with 1 μM D + 1 μM Q for 24 h; (4) D+Q, DOX; cells were treated with 100 nM doxorubicin for 24 h. Doxorubicin was washed out after 24 h and cells were incubated in normal medium for six additional days, before treatment with D+Q for additional 24 h (Figure 1A). To assess cellular senescence at the end of the treatments, C12FDG, a fluorogenic substrate for β-galactosidase, was used for detection of senescence associated (SA) β-gal positivity, a bona-fide standard marker of senescent cells. C12FDG retention within the cells was evidenced by the distribution plots obtained from the flow cytometer in the area with stronger green fluorescence intensity (Figure S1B). DOXO induced a 40- and 400-fold increase in SA-β-galactosidase activity in HepG2 and in Huh-7 cells, respectively (Figure 1B). Surprisingly D+Q treatment had no effects in removing β-gal positive cells upon DOX treatment, as well as D+Q alone had no significant effects compared to untreated cells (Figure 1B). Next, we quantified the activation of p16, p21, and γH2A.X, established markers of senescence and of DOX-induced DNA damage, respectively (19, 20), using immunofluorescence. As expected, DOX induced significant increase in the amount of nuclei with more than 5 γH2A.X distinct foci marking DNA lesions, and also in p16 and p21 staining, in Huh-7 cells (Figures 1C,D,S1C) and in HepG2 cells (Figures S2A–C). D+Q had no effect in preventing the activation of these markers of senescence upon DOX treatment (Figures 1C,D, S1C, S2A–C). In parallel we measured the expression levels of key genes involved in cellular senescence (p16, p21) and in SASP (IL-6, IL-8, MMP1, MMP3), using qPCR. DOX increased p21, IL-8, MMP1, MMP3, and TGF-β mRNA levels in both Huh-7 and HepG2 cells (Figures S1D, S2D). Interestingly, IL-6 mRNA was not expressed in HepG2 while it was found increased by DOX treatment in Huh-7 cells (Figure S1D). D+Q was largely ineffective in preventing the activation of senescence/SASP genes in both cell types upon DOX treatment (Figures S1D, S2D). In summary, DOX induced cellular senescence in HCC cells, manifested by the SA-β-gal positivity, flattened nuclear morphology, appearance of senescent markers and SASP gene expression; however D+Q did not exhibit senolytic activity in our *in vitro* experimental setup involving DOX-induced cellular senescence. To investigate the *in vivo* antitumor efficacy of doxorubicin, D+Q, or the combined treatment, xenograft studies were performed. Subcutaneous HCC xenografts from Huh-7 cells stably over-expressing a far-red fluorescent protein (eqFP650) were established on the dorsal flank of immunodeficient athymic nu/nu mice, and treated until tumor size in the control/untreated group reached 1,400 mm^3^ (~23 d post-inoculation). Four experimental groups of balb/c nude mice (*n* = 11 per group) implanted with Huh-7-eqFP650 were created as it follows: (1) CTL, control mice i.p. injected with vehicle alone (PBS); (2) DOX, mice injected with 4 mg/kg doxorubicin at days 7 and 14 post-implantation; (3) D+Q, mice administered with Dasatinib (D, 5 mg/kg) + Quercetin (Q, 50 mg/kg) by oral gavage, at days 9 and 16 post-implantation; (4) D+Q + DOX, mice injected with 4 mg/kg doxorubicin at days 7 and 14 post-implantation, and simultaneously administered with D+Q by oral gavage, at days 9 and 16 post-implantation (Figure 2A). Tumor volume measurement by caliper and eqFP650 *in vivo* imaging was performed every 2–3 days until euthanasia. Time-dependent tumor volume growth is illustrated in Figures 2B,C: average tumor volume in mice of group 3 (D+Q) exceeded of 50% the average tumor volume in mice of group 1 (CTL) (*p* = 0.0252). Treatment of doxorubicin reduced tumor growth of 30% (group 2 vs. group 1, *p* = 0.0486; Figure 2C). Synergistic treatment of mice with D+Q did not further enhance DOX-induced tumor growth inhibition (Figure 2C). Independent assessment of tumor volume using eqFP650-dependent body fluorescence imaging (Figure S3A) or explanted tumor weight (Figure S3B) at sacrifice largely mirrored the values of tumor growth (Figure 2C). Of note, D+Q treated-tumors showed increased proportion of fibrotic-like tissue upon H/E staining (Figure S3C). Overall these data indicate that D+Q is ineffective in clearing chemotherapy-induced HCC senescent cells as also shown on selected tumors sections stained for the SA-β-gal positivity (Figure 2D). Conversely, D+Q alone displayed pro-tumorigenic effects *in vitro* and *in vivo* in the absence of chemotherapy. This calls for concern about the utilization of senolytics and of Q, which is often taken as off-the-counter supplement for its documented anti-oxidant properties, in advanced liver diseases. Moreover, a recent clinical trial on D treatment of patients with advanced liver cancer that cannot be removed by surgery, showed no efficacy and significant side effects^1^. It remains to be demonstrated what impact of D+Q may have in dietary/genetic mice models of NAFLD slowly progressing into HCC (21). Whereas, D and/or Q treatment might exert a protective/therapeutic effect against NAFLD at high (9) but not low (22) doses, we demonstrate that these compounds are largely ineffective alone, or in synergy with senescence-inducing chemotherapy, against experimental HCC.

## Material and methods

### Cell cultures and treatment

HepG2 and Huh-7 parental lines were obtained from CLS-GmbH and cultured in DMEM (1X) supplemented with 10% fetal bovine serum (FBS), with 1% penicillin/streptomycin. Viral production and transduction of Huh-7 cells to stably express RFP was previously described (7, 23). For chemotherapy-inducing senescence experiments, HepG2 and Huh-7 cells were treated for 24 h with 100 nM Doxorubicin (Sigma-Aldrich) as vehicle DMSO was used. Cell viability was assessed by using alamarBlue™ Cell Viability Reagent (ThermoScientific), according to manufacturer's instructions.

### Generation of stable eqFP650-Huh-7 clonal cell line

Stable Huh-7 clonal cell line expressing the far-red fluorescent protein eqFP650 was derived via lentiviral transduction. The cDNA encoding eqFP650 (17) was placed into the HIV1-based self-inactivating lentiviral vector under the control of human EF1α-promoter. The puromycin resistance gene is linked via an internal ribosomal entry site (IRES) element [as described in (8)]. Virus was produced by transient transfection in HEK293T cells and used to transduce Huh-7 cells with MOI > 10 for 48 h in the presence of 4 μg/mL Polybrene. Cells were grown for a week under 3 μg/mL puromycin selection, expanded and used to produce subcutaneous xenograft tumors in immunodeficient mice. Cells from tumor biopsy were re-derived into culture, and plated to multiwell plates at limited dilution. Positive single-cell derived clones were characterized, and a high-expressing clone designated A1 was used in subsequent experiments.

### Mouse xenografts

The study was conducted in the Animal Facility of Tallinn University of Technology, in accordance with European Directive 2010/63/EU and National Animal Welfare legislation; the experimental protocol was approved by the ethical committee of Estonian Ministry of Agriculture (now Ministry of Rural Affairs). Forty immunodeficient athymic *nu/nu* mice (Envigo) were injected subcutaneously with 5 × 10^6^ of exponentially growing eqFP650-Huh-7 (clone A1) cells per animal, in 100 μl 50% media: Matrigel mixture. For treatment, mice were randomized into four cohorts: vehicle alone; doxorubicin; D+Q; and doxorubicin combined with D+Q. Drugs were dissolved in DMSO (doxorubicin at 10 mg/ml; D+Q in a single stock solution of 5 and 50 mg/ml, respectively) and dilutions were made just prior to the treatment procedure. Doxorubicin was diluted in PBS and injected i.p. at 4 mg/kg body weight, D+Q stock was diluted 1/10 in water and administered by oral gavage at 5 and 50 mg/kg body weight, respectively (As D+Q precipitates in aqueous solutions, dilution was prepared individually for each mouse, and the whole contents of the tube was gavaged). Twice a week, images of isoflurane-sedated mice were acquired using IVIS Lumina (PerkinElmer) with 7.5 × 7.5 cm field-of-view, medium binning, 1–2 s exposure time and 500/Cy5.5, 535/Cy5.5, 570/Cy5.5, 605/Cy5.5 excitation (nm)/emission filter pairs. Images were analyzed with LivingImage (PerkinElmer). Fluorescence data were reported as background-subtracted signal (Signal–Bkg), where background fluorescence was measured from the same mouse nearby the injection site, as described (Sci Rep 10332). Tumor volume measurements were performed by a digital caliper in two dimensions, length (L) and width (W), and volume was calculated according to the formula V = L × W^2^/2.

### CF_12_FDG staining

Cellular senescence was quantified using CF_12_FDG [5-Dodecanoylaminofluorescein Di-β-D-Galactopyranoside] (Satereh Biotech). CF_12_FDG is a non-fluorescent, lipophilic, beta-galactosidase substrate. The substrate is cleaved by ß-galactosidase producing a fluorescent product that is well-retained by the cells. CF_12_FDG detection in HepG2 and Huh-7 cells was performed by flow cytometry as it is described in (24). Briefly, lysosomal alkalinization of HepG2 or Huh-7 cells was induced by incubation of Baf A1 (100 nM) in humidified air with 5% CO_2_ at 37 C for 1 h. CF_12_FDG was added in the culture media containing Baf A1 for another 2 h. After fixation with 4% formaldehyde at room temperature, nuclei were stained with DAPI. To quantify the cells positive for CF_12_FDG, cells were rinsed with PBS, trypsinized, collected, and resuspended in ice cold PBS. The HepG2 hepatocyte suspension was analyzed using a flow cytometer FACSCanto (BD Biosciences).

### qPCR

Total RNA was extracted from HepG2 and Huh-7 cells using Trizol® (Invitrogen), according to manufacturer's instructions. Real Time-PCR was performed in triplicate utilizing StepOnePlus™ Real-Time PCR System (Applied Biosystems, Darmstadt, Germany) and SYBR™ Select Master Mix (ThermoScientific).

Human primer sequences were as it follows:

p16 Forward 5′-ATGGAGCCTTCGGCTGACT-3′ Reverse 5′-GTAACTATTCGGTGCGTTGGG-3′; p21 Forward 5′-TGTCCGTCAGAACCCATGC-3′ Reverse 5′-AAAGTCGAAGTTCCATCGCTC-3′ IL-6 Forward 5′-ACTCACCTCTTCAGAACGAATTG-3′, Reverse 5′-CCATCTTTGGAAGGTTCAGGTTG-3′; IL-8 Forward 5′-ACTGAGAGTGATTGAGAGTGGAC-3′, Reverse 5′-AACCCTCTGCACCCAGTTTTC-3′; TGF-β, Forward 5′-GGCCAGATCCTGTCCAAGC-3′, Reverse 5′-GTGGGTTTCCACCATTAGCAC-3′.

MMP1 Forward 5′-CTCTGGAGTAATGTCACACCTCT-3′ Reverse 5′-TGTTGGTCCACCTTTCATCTTC-3′; MMP3 Forward 5′-CTGGACTCCGACACTCTGGA-3′, Reverse 5′-CAGGAAAGGTTCTGAAGTGACC-3′; RPLP0 Forward 5′-CTGGAAGTCCAACTACTTCCT-3′, Reverse 5′-CATCATGGTGTTCTTGCCCAT-3′; GAPDH Forward 5′-GGATTTGGTCGTATTGGG-3′, Reverse 5′-GGAAGATGGTGATGGGATT-3′.

### Immunofluorescence

Immunofluorescence in HCC cells was performed as previously described (25). Briefly, cells were fixed in 4% paraformaldehyde for 20 min and permeabilized with 0.1% Triton X-100 (Sigma-Aldrich). 1:1,000 primary antibody was incubated overnight at 4°C. Primary antibodies were from Abcam (gammaH2A.X, CDKN2A/p16INK4a, p21). The staining was developed using Alexa fluorescent (488, 555) conjugated secondary antibodies, and images were acquired using a Axio scan Z.1 or LSM 7 DUO microscopy system, respectively (Zeiss) (LeicaMicrosystems, Wetzlar, Germany).

### Immunohistochemistry

Sections from xenograft Huh-7 liver tumors specimens were processed by hematoxylin and eosin staining for histologic evaluation. Immunostaining was performed using the iVIEW DAB Detection Kit. Primary antibody for anti-GLB1/Beta-Galactosidase Antibody (clone 5H2) IHC-plus LS-B10217 was obtained from LSBio. Anti-GLB1 primary antibody was diluted 1:100. Positive and negative controls were run concurrently. Means of triplicate counts were used for statistical analyses. The percentage of brown staining, indicating β-galactosidase positivity, was calculated in 10 random high-power field (HPF) at 200× magnification and expressed as means. All analyses were performed in triplicate by two independent pathologists.

### Statistical tests

Results are expressed as means ± s.d. Comparisons between groups were performed with the parametric Student's *t*-test or the non-parametric Mann–Whitney U-test, as appropriate, using GraphPad Prism Software (version 5.00 for Windows, San Diego, CA, USA): a *P*-value ≤ 0.05 was considered significant.

## Author contributions

KK, MS, MH, PP, and IP: acquisition, analysis, and interpretation of data. MM and JB: critical revision of the manuscript for important intellectual content. MV conceived and designed the study, obtained funding and drafted the manuscript. All authors critically revised the manuscript and approved its final version.

### Conflict of interest statement

The authors declare that the research was conducted in the absence of any commercial or financial relationships that could be construed as a potential conflict of interest.

## Footnotes

^1^Available online at: https://clinicaltrials.gov/ct2/show/record/NCT00459108?sect=X30156.

## Supplementary material

Figure S1 Effect of D+Q on chemotherapy-induced senescence of HCC cells. **(A)** Dose-response of D or Q on the cell viability. Cells were incubated for 24 h with increasing concentrations (0-1-5 nM, 10-100-500 nM, 1-5-10-50-100-500 μM, 1 mM) of either drug, before viability assay. **(B)** Representative fluorescence scatter plot of SA-β-gal in HuH-7 cells, control or DOX-treated. **(C)** Quantification of p21 staining intensity. **(D)** qPCR measurement of mRNA levels of cellular senescence (p16, p21) and SASP (IL-8, MMP1, MMP3) factors in HuH-7 cells. Results are expressed as fold induction relative to control, following normalization to expression of the housekeeping gene phosphoribosomal protein P0 (RPLP0) and GAPDH. ^*^*p* < 0.05; ^**^*p* < 0.01; ^***^*p* < 0.001 compared to CTL.

Figure S2 Effect of D+Q on chemotherapy-induced senescence of HCC cells. **(A)** Representative immunofluorescence micrographs display staining for DAPI, p16 and γ-H2A.X in control, DOX-treated, D+Q-treated or D+Q, DOX-treated HepG2 cells. **(B)** Quantification of p16 staining intensity or of γ-H2A.X positive cells. For the latter staining, cells with five or more nuclear foci were scored as positive. Approximately 600 cells per group were counted. **(C)** Quantification of p21 staining intensity. **(D)** qPCR measurement of mRNA levels of cellular senescence and SASP factors in HepG2 cells. Results are expressed as fold induction relative to control, following normalization to RPLP0 and GAPDH. ^*^*p* < 0.05; ^**^*p* < 0.01; ^***^p < 0.001 compared to CTL.

Figure S3 Effect of D+Q on chemotherapy-induced decrease in tumor growth. **(A)** Huh-7 cells stably expressing RFP were imaged using IVIS Lumina II. Left panel: representative image of a tumor-engrafted mouse at 21 days. Right panel: image-assisted quantification of tumor fluorescence intensity in mice in CTL (*n* = 3), DOX (*n* = 10), D+Q (*n* = 9), D+Q, DOX (*n* = 7). **(B)** At sacrifice, tumors were excised and weighted. *N* = 11 per group. **(C)** Representative pictures of eosin and SA-β-gal immunostaining of tumor sections from mice as in Figure 2 (*n* = 3). ^*^*p* < 0.05; ^**^*p* < 0.01 compared to CTL.
