# Supplementary figures and images for: Senolytic Cocktail Dasatinib+Quercetin (D+Q) Does Not Enhance the Efficacy of Senescence-Inducing Chemotherapy in Liver Cancer
Source: Front Oncol. 2018 Oct 30;8:459. doi: 10.3389/fonc.2018.00459 (PMC6218402; doi:10.3389/fonc.2018.00459)

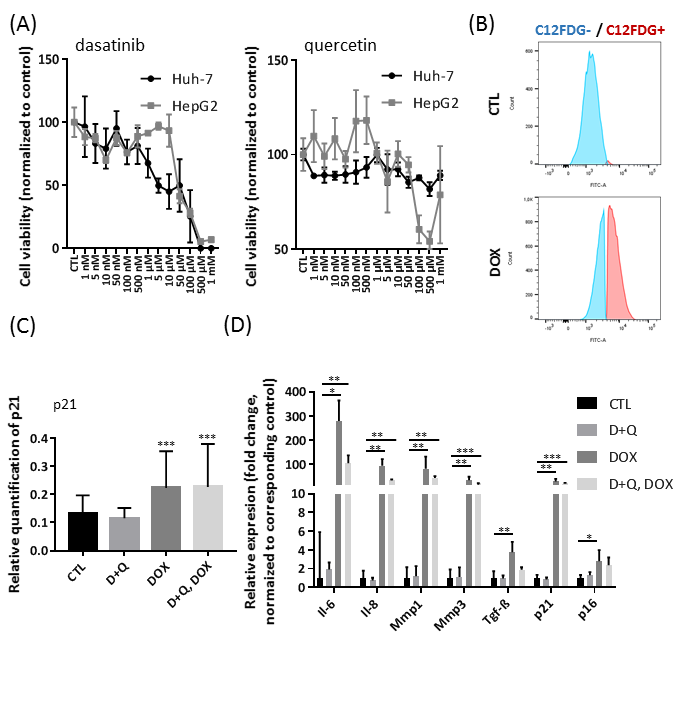

Supplement: Figure S1 — Effect of D+Q on chemotherapy-induced senescence of HCC cells. (A) Dose-response of D or Q on the cell viability. Cells were incubated for 24 h with increasing concentrations (0-1-5 nM, 10-100-500 nM, 1-5-10-50-100-500 μM, 1 mM) of either drug, before viability assay. (B) Representative fluorescence scatter plot of SA-β-gal in HuH-7 cells, control or DOX-treated. (C) Quantification of p21 staining intensity. (D) qPCR measurement of mRNA levels of cellular senescence (p16, p21) and SASP (IL-8, MMP1, MMP3) factors in HuH-7 cells. Results are expressed as fold induction relative to control, following normalization to expression of the housekeeping gene phosphoribosomal protein P0 (RPLP0) and GAPDH. *p < 0.05; **p < 0.01; ***p < 0.001 compared to CTL. [file Image_1.TIF]

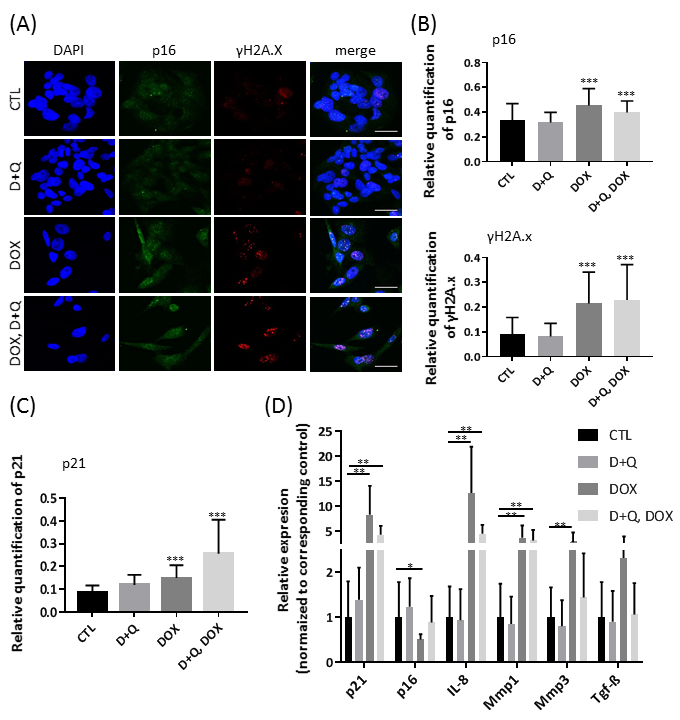

Supplement: Figure S2 — Effect of D+Q on chemotherapy-induced senescence of HCC cells. (A) Representative immunofluorescence micrographs display staining for DAPI, p16 and γ-H2A.X in control, DOX-treated, D+Q-treated or D+Q, DOX-treated HepG2 cells. (B) Quantification of p16 staining intensity or of γ-H2A.X positive cells. For the latter staining, cells with five or more nuclear foci were scored as positive. Approximately 600 cells per group were counted. (C) Quantification of p21 staining intensity. (D) qPCR measurement of mRNA levels of cellular senescence and SASP factors in HepG2 cells. Results are expressed as fold induction relative to control, following normalization to RPLP0 and GAPDH. *p < 0.05; **p < 0.01; ***p < 0.001 compared to CTL. [file Image_2.TIF]

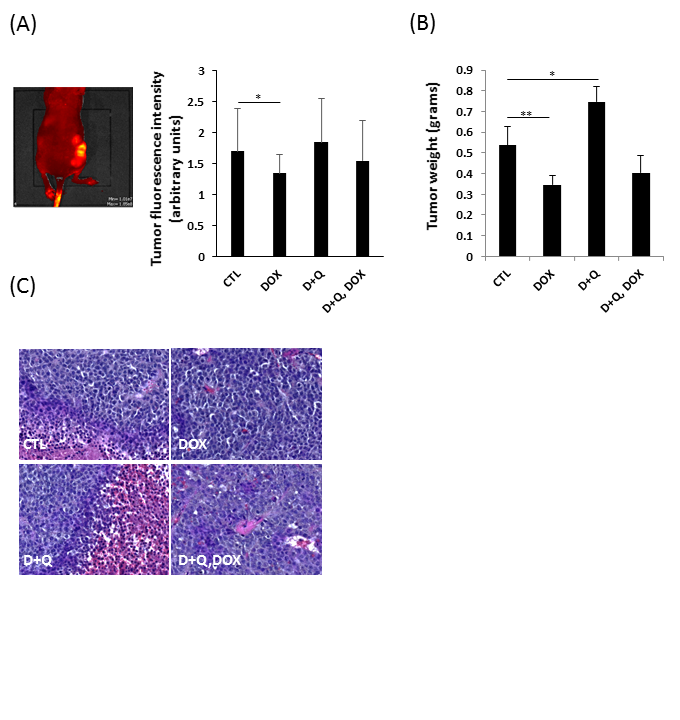

Supplement: Figure S3 — Effect of D+Q on chemotherapy-induced decrease in tumor growth. (A) Huh-7 cells stably expressing RFP were imaged using IVIS Lumina II. Left panel: representative image of a tumor-engrafted mouse at 21 days. Right panel: image-assisted quantification of tumor fluorescence intensity in mice in CTL (n = 3), DOX (n = 10), D+Q (n = 9), D+Q, DOX (n = 7). (B) At sacrifice, tumors were excised and weighted. N = 11 per group. (C) Representative pictures of eosin and SA-β-gal immunostaining of tumor sections from mice as in Figure 2 (n = 3). *p < 0.05; **p < 0.01 compared to CTL. [file Image_3.TIF]
